# Supplementary figures and images for: DAF-18/PTEN protects LIN-35/Rb from CLP-1/CAPN-mediated cleavage to promote starvation resistance
Source: Life Sci Alliance. 2025 Apr 8;8(6):e202403147. doi: 10.26508/lsa.202403147 (PMC11979363; doi:10.26508/lsa.202403147)

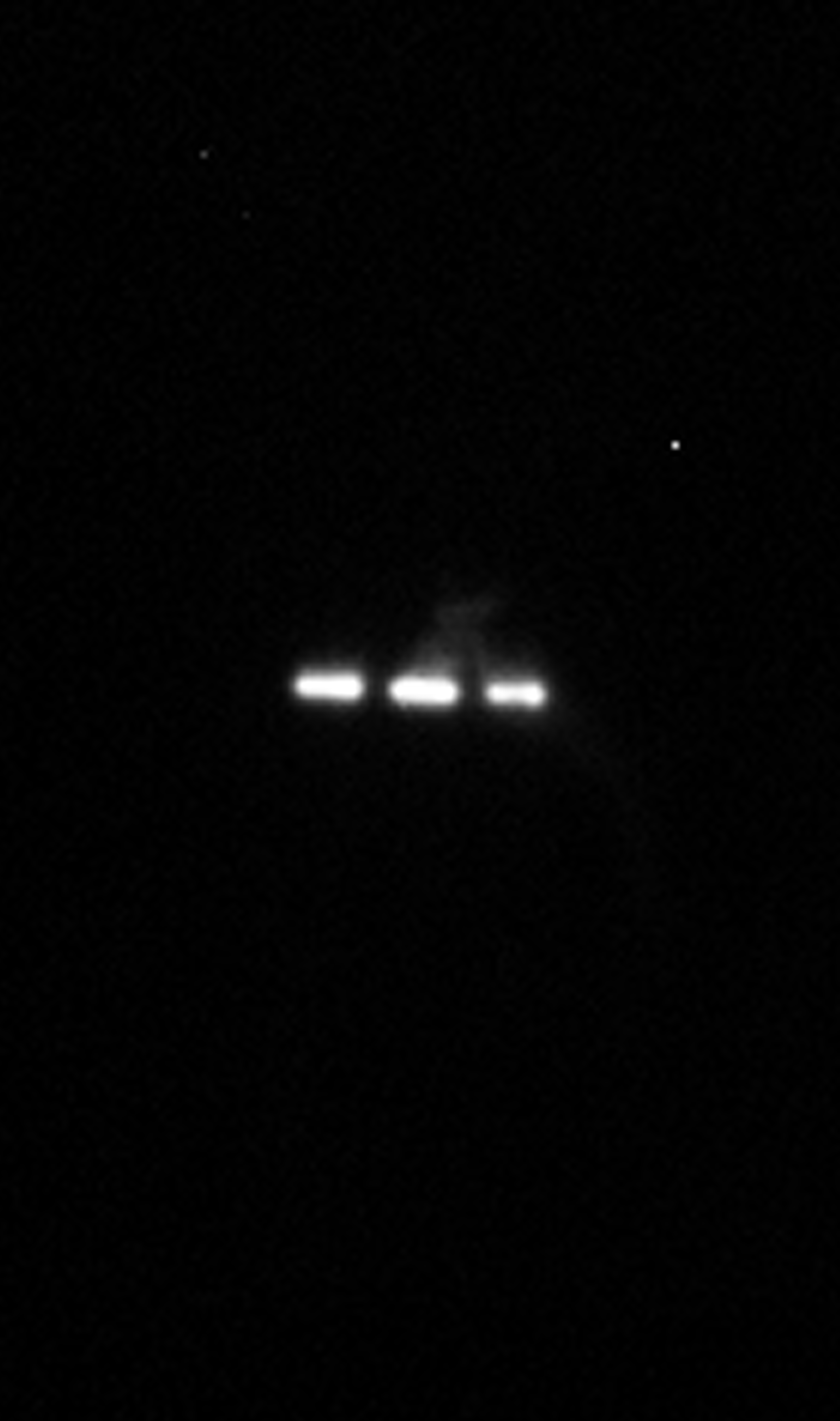

Supplement: Supplementary file 3 [file LSA-2024-03147_SdataF4_F5_FS2.zip › S1 File/western_blot_images_for_Fig4AandB/rep1_anti_alpha_tubulin.tif]

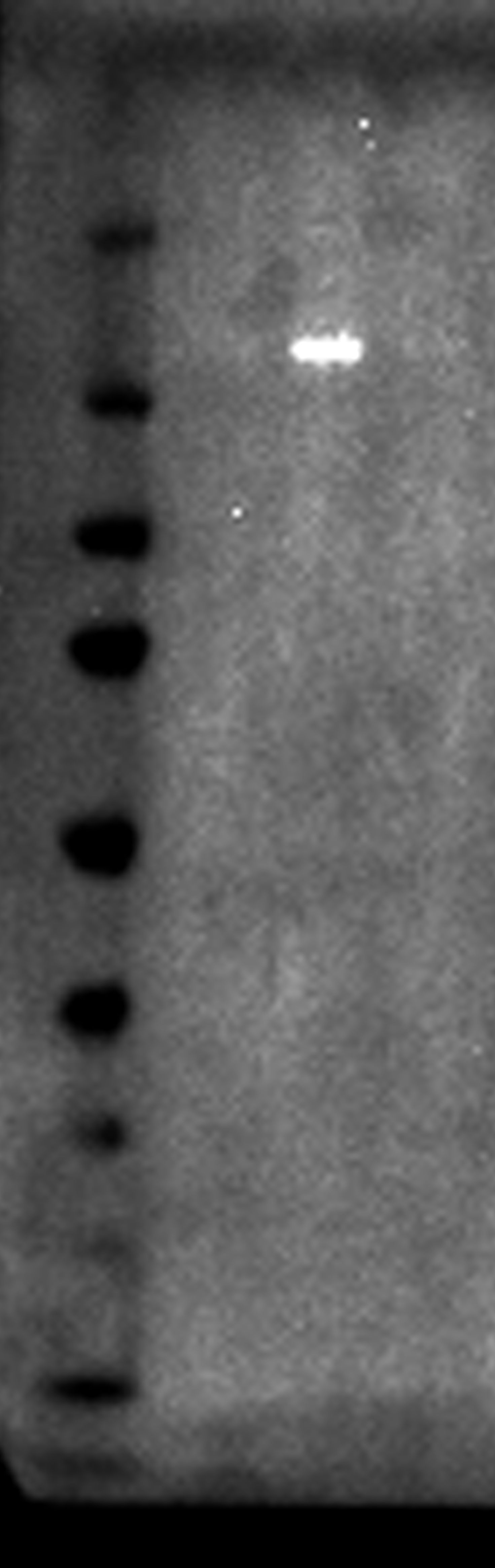

Supplement: Supplementary file 3 [file LSA-2024-03147_SdataF4_F5_FS2.zip › S1 File/western_blot_images_for_Fig4AandB/rep1_anti_GFP.tif]

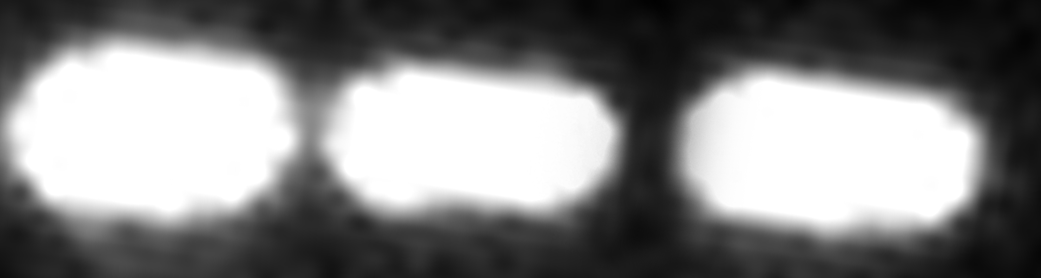

Supplement: Supplementary file 3 [file LSA-2024-03147_SdataF4_F5_FS2.zip › S1 File/western_blot_images_for_Fig4AandB/rep2_anti_alpha_tubulin.tif]

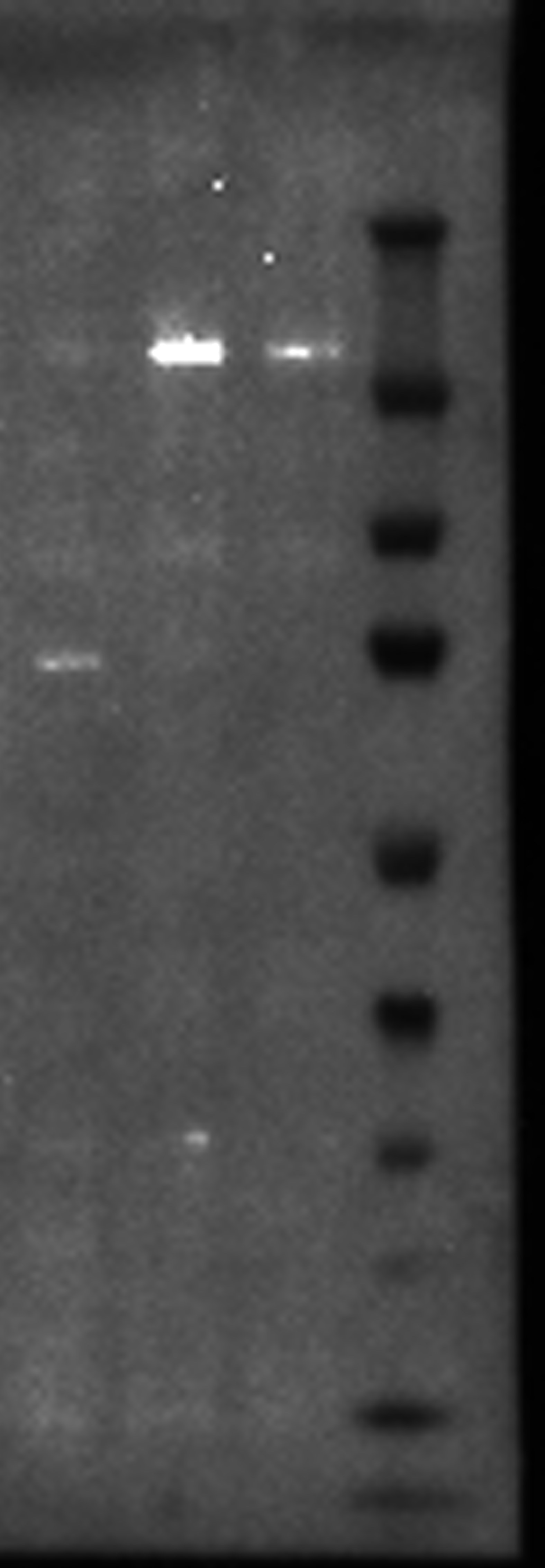

Supplement: Supplementary file 3 [file LSA-2024-03147_SdataF4_F5_FS2.zip › S1 File/western_blot_images_for_Fig4AandB/rep2_anti_GFP.tif]

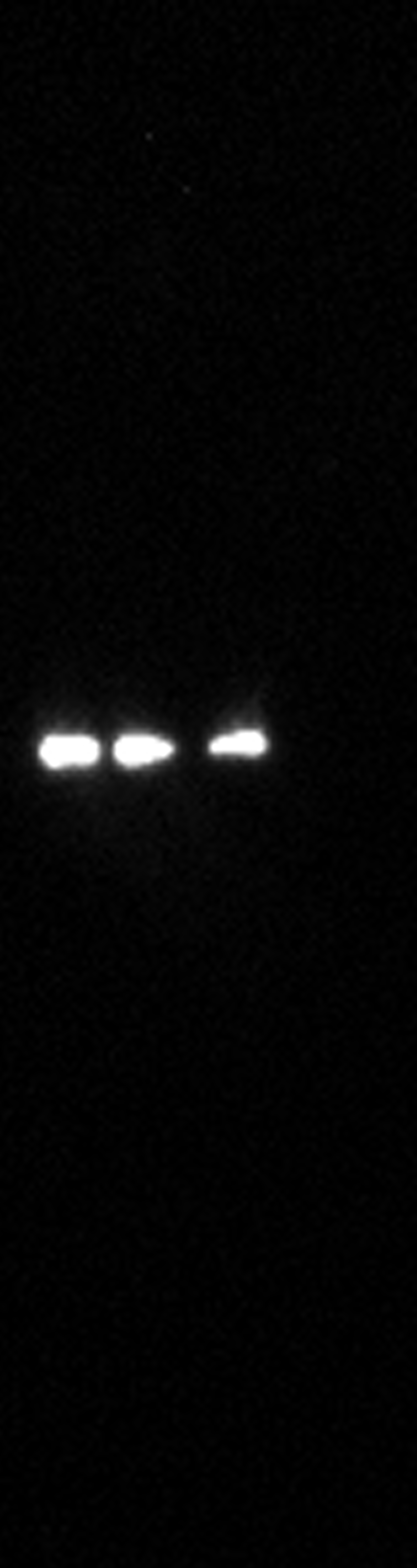

Supplement: Supplementary file 3 [file LSA-2024-03147_SdataF4_F5_FS2.zip › S1 File/western_blot_images_for_Fig4AandB/rep3_anti_alpha_tubulin.tif]

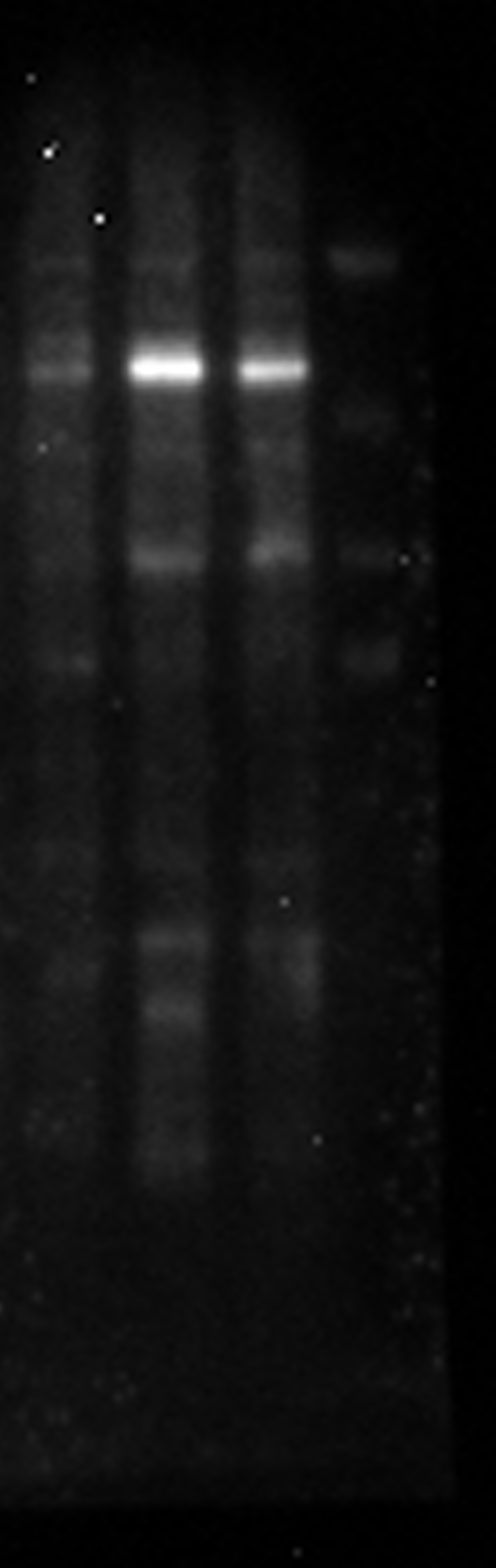

Supplement: Supplementary file 3 [file LSA-2024-03147_SdataF4_F5_FS2.zip › S1 File/western_blot_images_for_Fig4AandB/rep3_anti_GFP.tif]

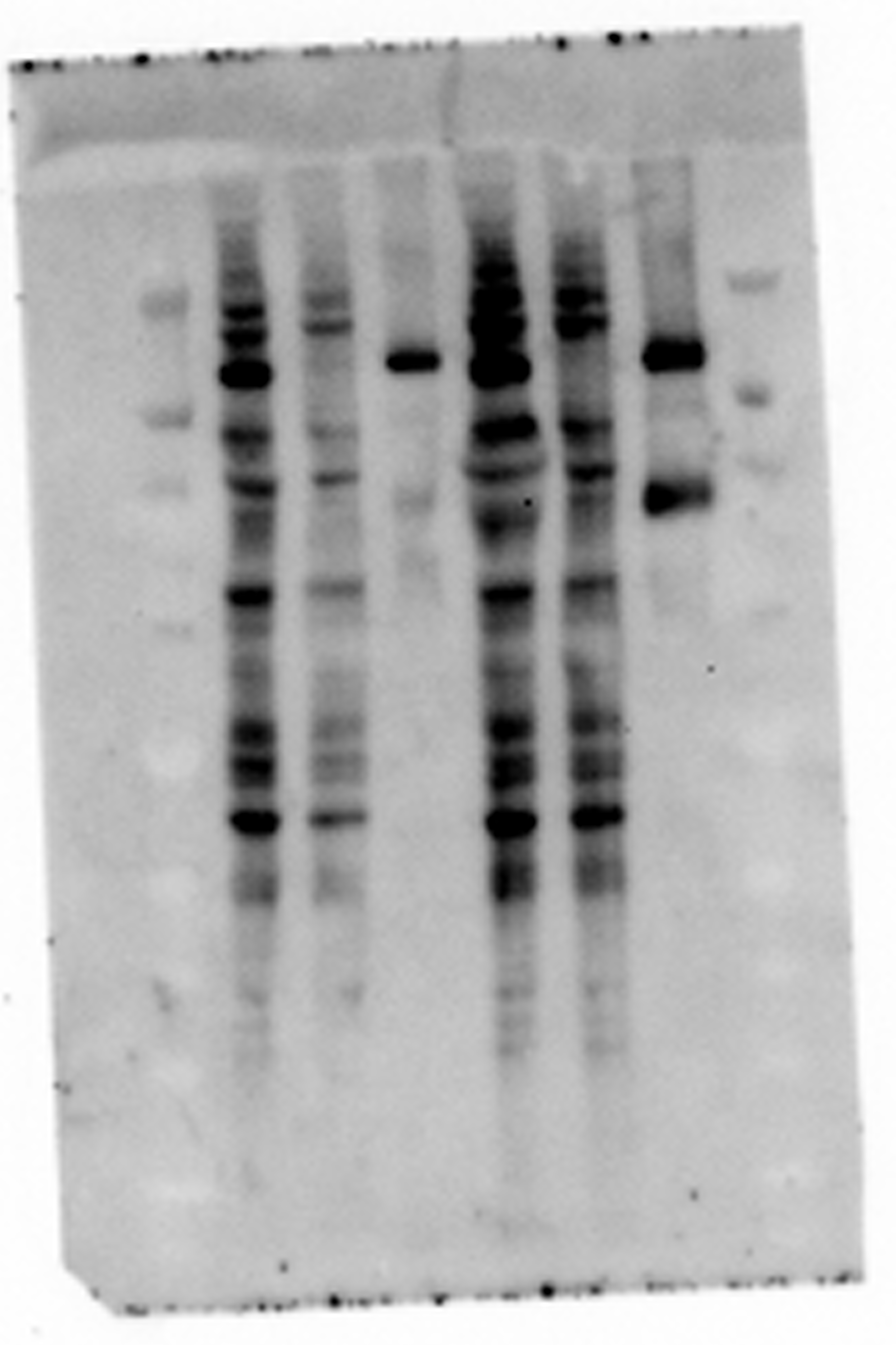

Supplement: Supplementary file 3 [file LSA-2024-03147_SdataF4_F5_FS2.zip › S1 File/western_blot_images_for_Fig4CandD/anti_GFP_rep1.tif]

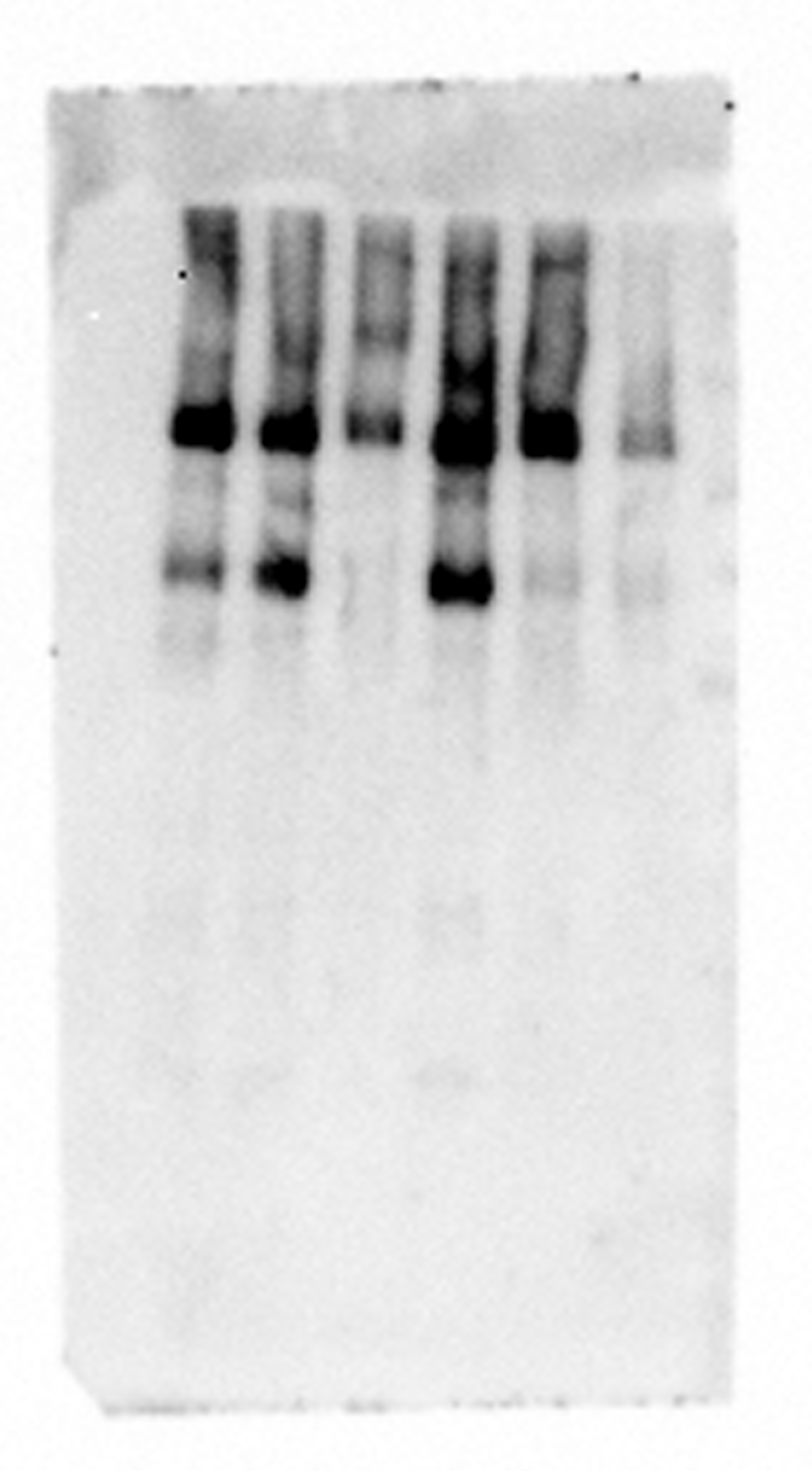

Supplement: Supplementary file 3 [file LSA-2024-03147_SdataF4_F5_FS2.zip › S1 File/western_blot_images_for_Fig4CandD/anti_GFP_reps2-4.tif]

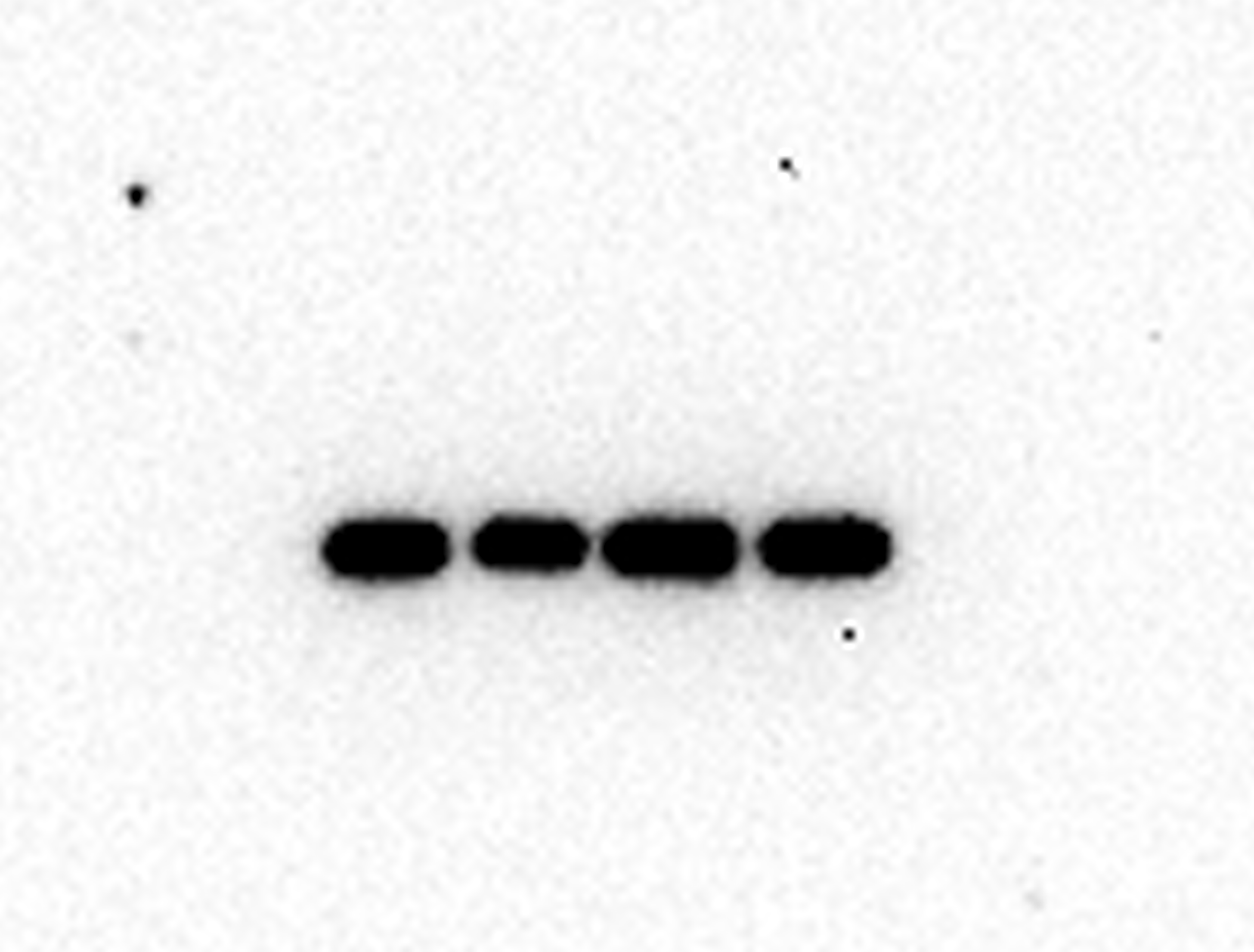

Supplement: Supplementary file 3 [file LSA-2024-03147_SdataF4_F5_FS2.zip › S1 File/western_blot_images_for_Fig5BandC/rep1_anti_alpha_tubulin.tif]

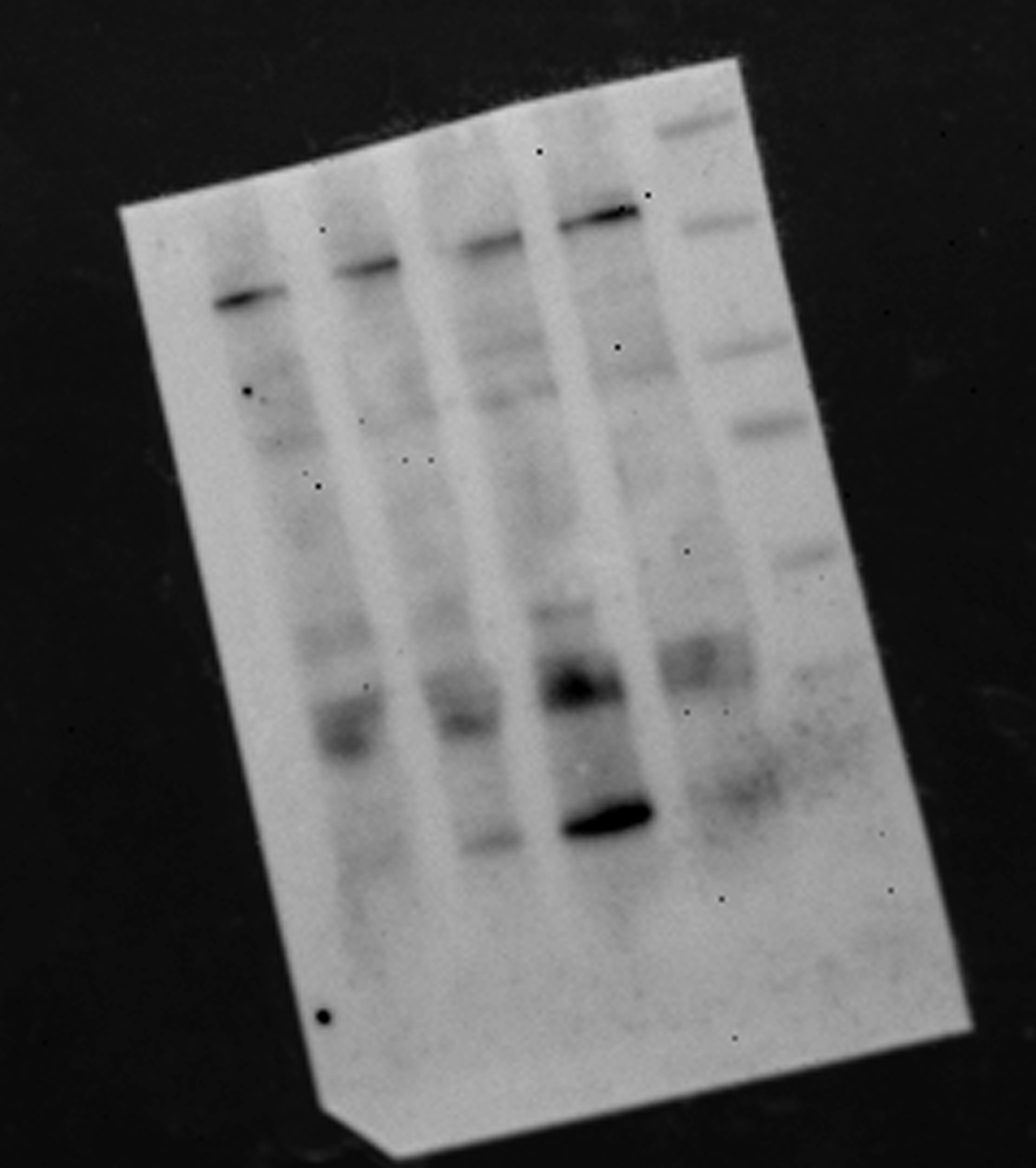

Supplement: Supplementary file 3 [file LSA-2024-03147_SdataF4_F5_FS2.zip › S1 File/western_blot_images_for_Fig5BandC/rep1_anti_GFP.tif]

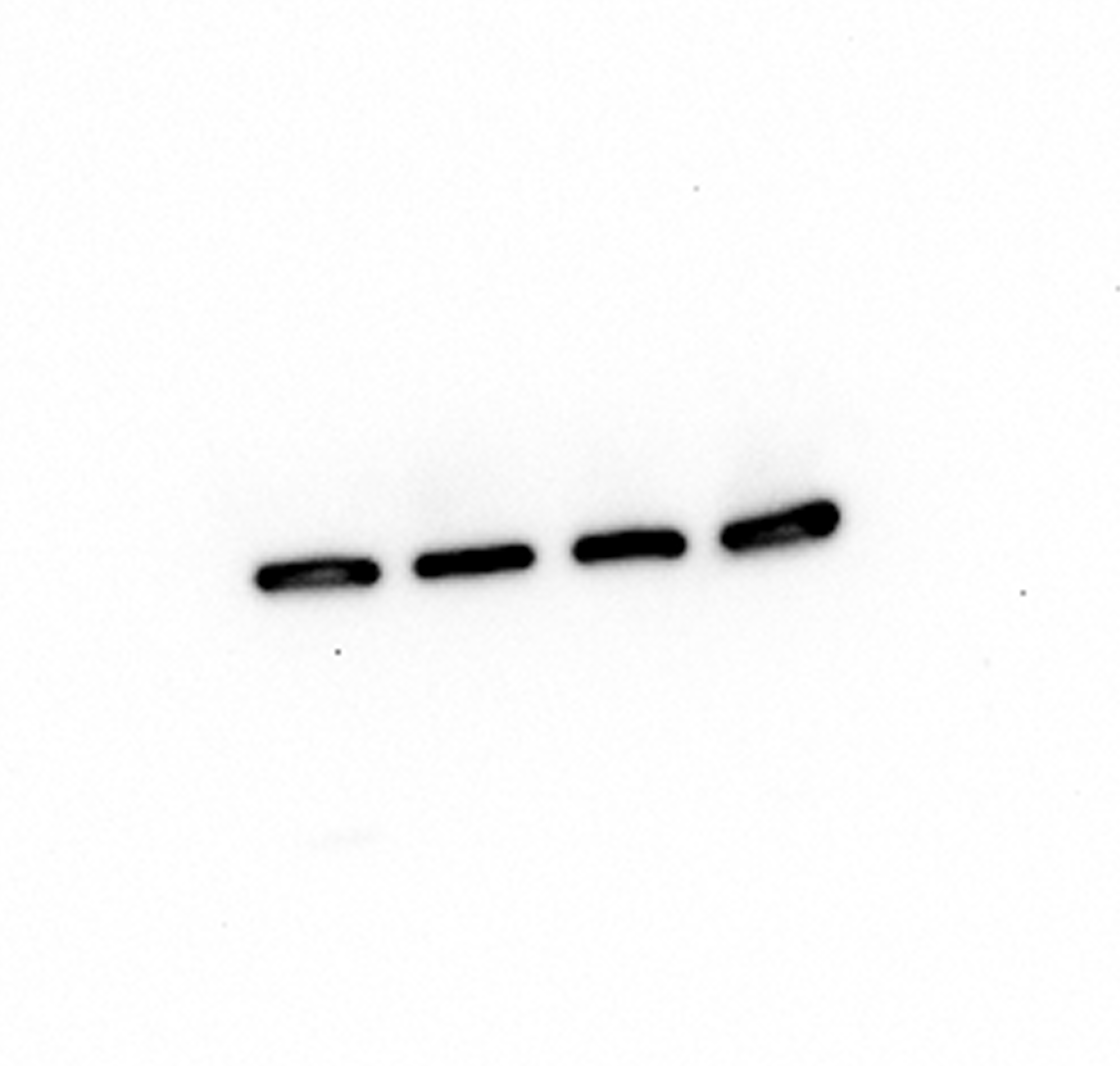

Supplement: Supplementary file 3 [file LSA-2024-03147_SdataF4_F5_FS2.zip › S1 File/western_blot_images_for_Fig5BandC/rep2_anti_alpha_tubulin.tif]

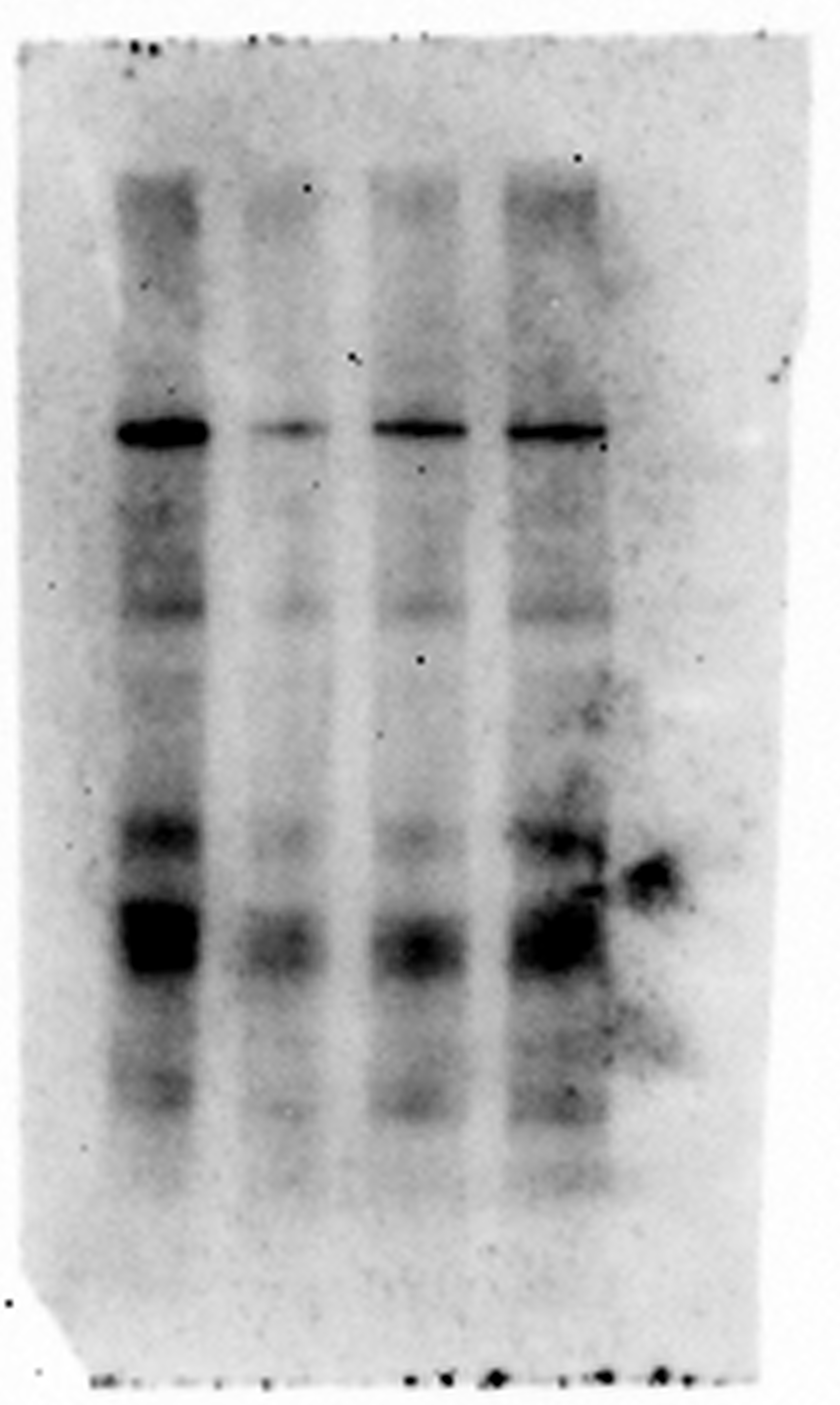

Supplement: Supplementary file 3 [file LSA-2024-03147_SdataF4_F5_FS2.zip › S1 File/western_blot_images_for_Fig5BandC/rep2_anti_GFP.tif]

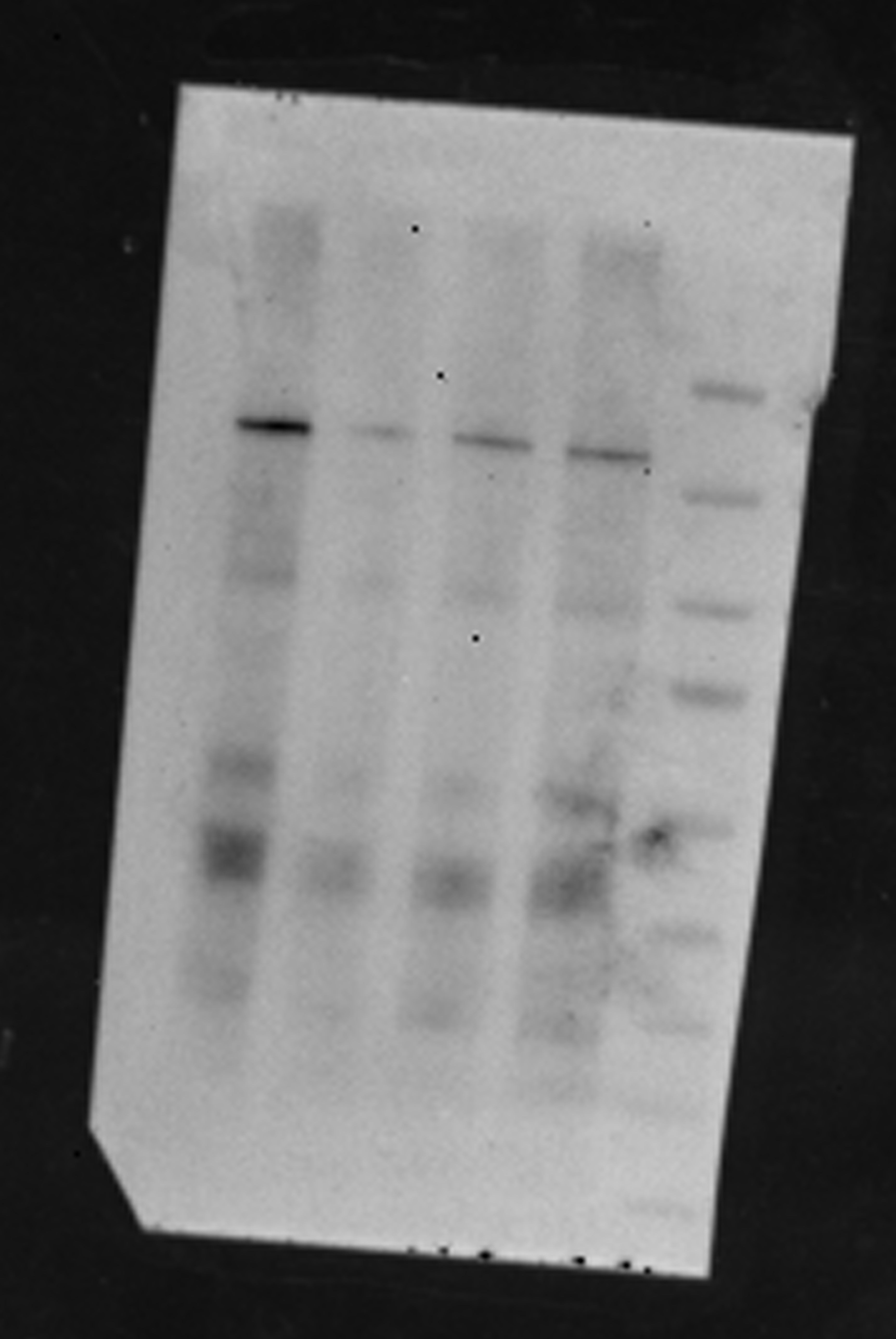

Supplement: Supplementary file 3 [file LSA-2024-03147_SdataF4_F5_FS2.zip › S1 File/western_blot_images_for_Fig5BandC/rep2_anti_GFP_showing_ladder.tif]

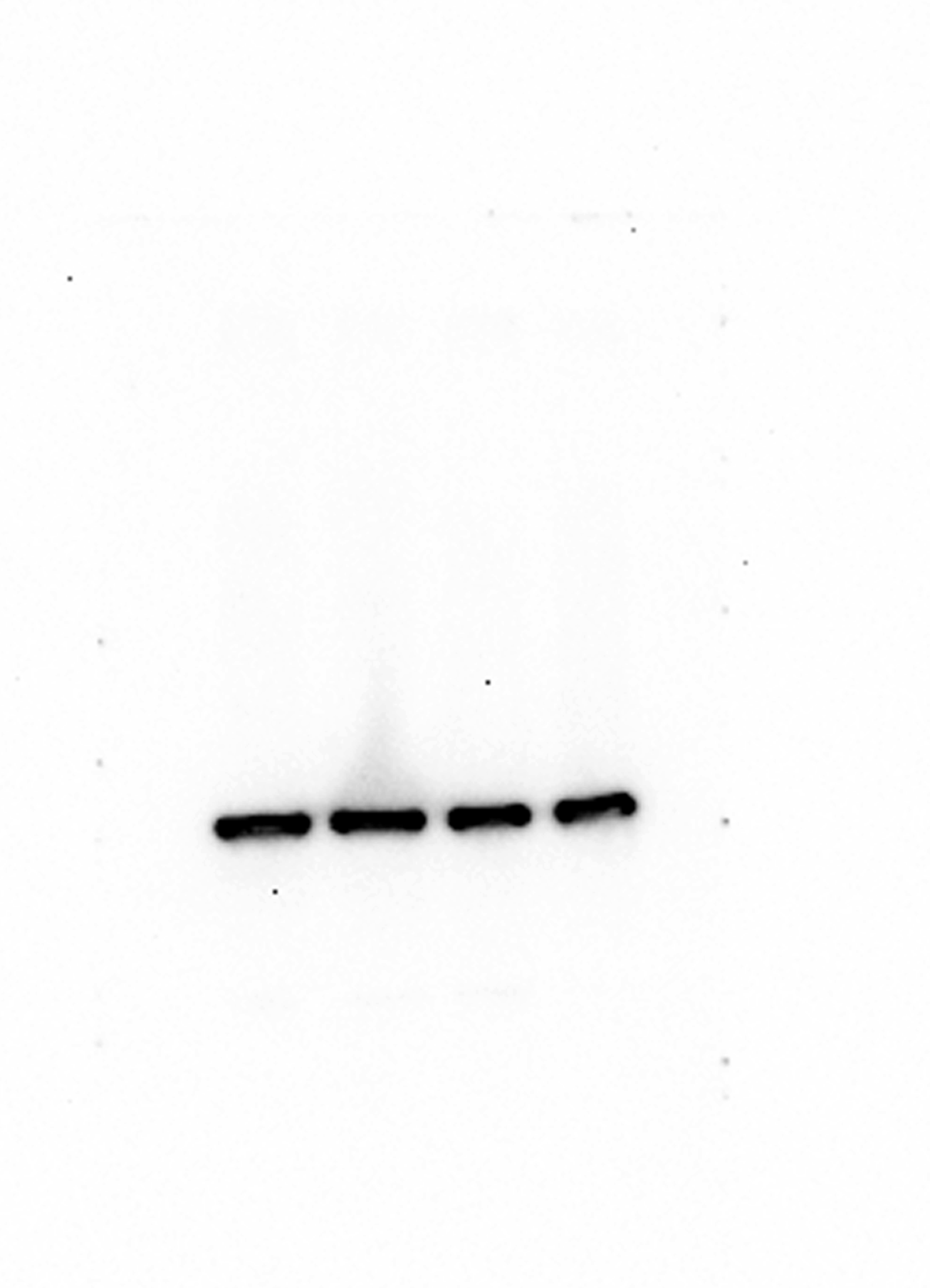

Supplement: Supplementary file 3 [file LSA-2024-03147_SdataF4_F5_FS2.zip › S1 File/western_blot_images_for_Fig5BandC/rep3_anti_alpha_tubulin.tif]

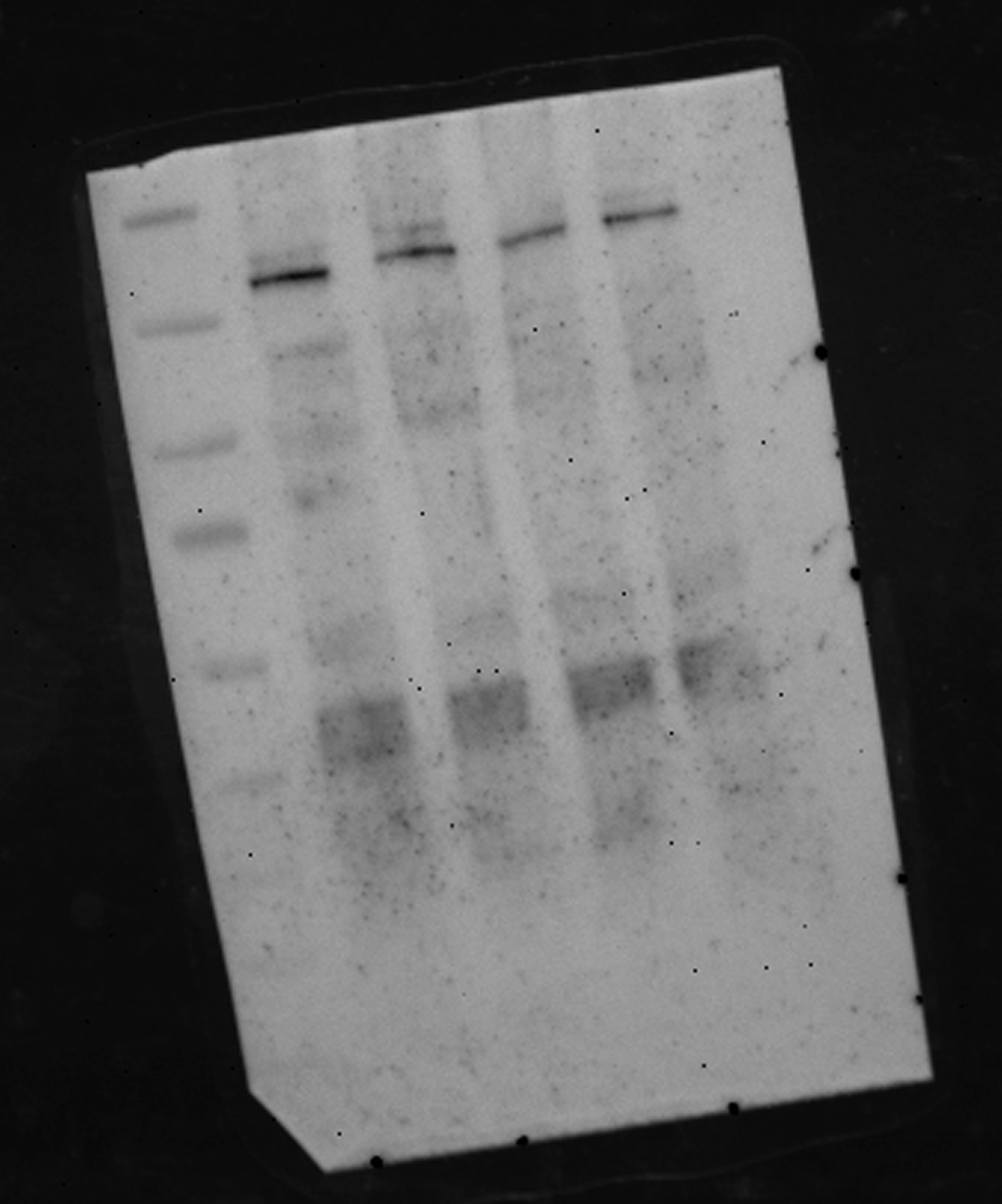

Supplement: Supplementary file 3 [file LSA-2024-03147_SdataF4_F5_FS2.zip › S1 File/western_blot_images_for_Fig5BandC/rep3_anti_GFP.tif]

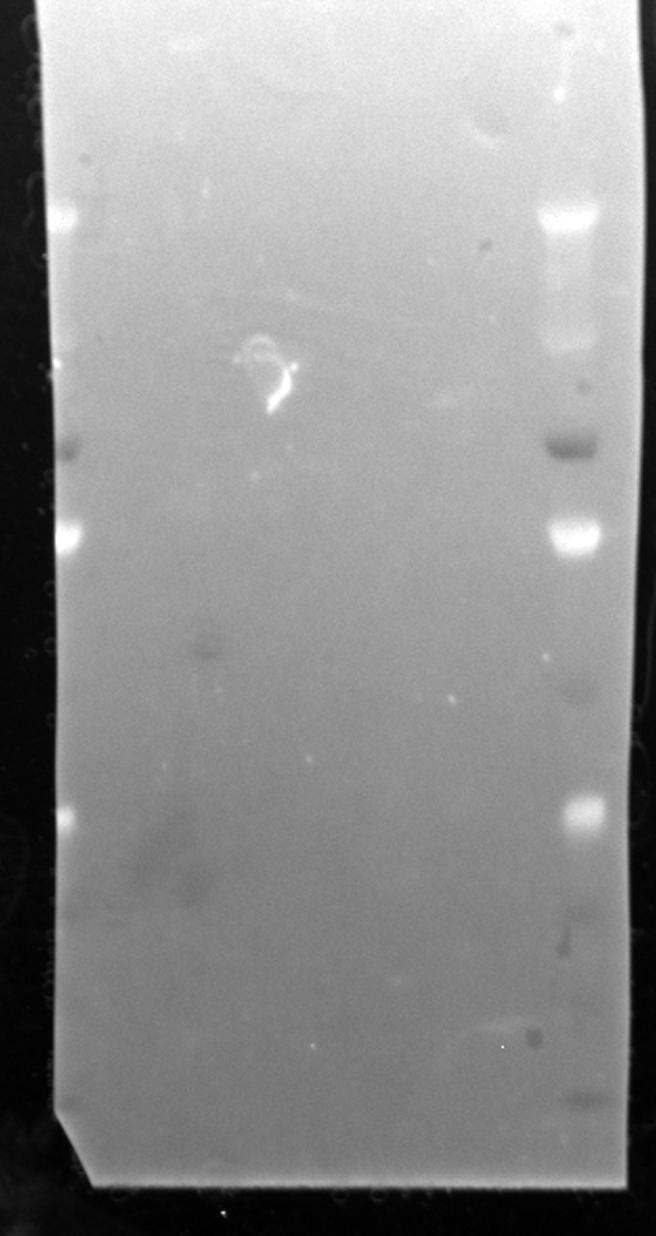

Supplement: Supplementary file 3 [file LSA-2024-03147_SdataF4_F5_FS2.zip › S1 File/western_blot_images_for_FigS2/rep2_membrane_with_ladder.tif]
